# Blockage of Conformational Changes of Heat Shock Protein gp96 on Cell Membrane by a α-Helix Peptide Inhibits HER2 Dimerization and Signaling in Breast Cancer

**DOI:** 10.1371/journal.pone.0124647

**Published:** 2015-04-21

**Authors:** Xin Li, Baozhong Wang, Weiwei Liu, Mingming Gui, Zheng Peng, Songdong Meng

**Affiliations:** 1 CAS Key Laboratory of Pathogenic Microbiology and Immunology, Institute of Microbiology, Chinese Academy of Sciences (CAS), Beijing, China; 2 University of Chinese Academy of Sciences, Beijing, China; 3 School of Life Sciences, Anhui University, Hefei, China; 4 Chinese People’s Liberation Army General Hospital, Beijing, China; University of South Alabama, UNITED STATES

## Abstract

Cell membrane translocation of heat shock protein gp96 from the endoplasmic reticulum has been observed in multiple tumors and is associated with tumor malignancy. However, the cancer-intrinsic function and the related mechanism of cell membrane gp96 as a pro-oncogenic chaperone remain further elucidated. In this study, we found that inhibition of gp96 intramolecular conformational changes by a single α-helix peptide p37 dramatically increased its binding to HER2, whereas decreased HER2 dimerization, phosphorylation and downstream signaling. Targeting cell membrane gp96 promoted HER2 ubiquitination and subsequent lysosomal degradation, which led to decreased cell growth and increased apoptosis, and inhibited tumor growth *in vivo*. We also demonstrate that gp96 inhibitory peptide p37 synergized with trastuzumab to suppress cell growth and induce apoptosis. Our work demonstrates that blocking gp96 conformational changes directs HER2 for cellular degradation, and represents a new therapeutic strategy for inhibiting HER2 signaling in cancer.

## Introduction

The heat shock protein 90 (HSP90) family contains four members in humans, HSP90α, HSP90β, gp96 (grp94) and Trap-1. As a chaperon in the endoplasmic reticulum (ER), gp96, together with other chaperons on the cytosolic side and ER side, constitutes a relay line for loading the cellular degraded peptides onto the MHC molecules in a concerted manner and activates specific T cell responses [1,2]. Meanwhile, gp96 associates with the newly synthesized polypeptides and guides their maturation and assembly in the secretory pathway. Until now a handful of gp96 client proteins have been identified which proper biosynthesis is dependent on gp96, including certain Toll-like receptors [3,4], integrins [5], the platelet glycoprotein Ib-IX-V complex [6], and immunoglobulin heavy chain [7], etc. Importantly, gp96 knockout mice in liver were found to be more susceptible to carcinogen-induced hepatocyte carcinogenesis [8]. These studies demonstrate that gp96 plays important roles in ER homeostasis and various physiological processes, as well as in human diseases, such as cancer.

The normally ER-resident gp96 may translocate to the cell membrane in some types of tumor cells and is associated with tumor malignancy. Cell membrane gp96 binds to HER2 [9–11], and metalloprotease pro-a disintegrin-like and metalloproteinase domain with thrombospondin type 1 motifs 9 (pro-ADAMTS9) [12]. The interaction between gp96 and its substrates sustains the stability or modulate the processing of these pro-oncogenic molecules, leading to increased tumor growth and angiogenesis. gp96 is known to shift between inactive and active conformations and associates with its client proteins in an ATP-sensitive manner [7,13], indicating that conformational changes of gp96 are needed for gp96 to guide its client proteins for proper biosynthesis. However, the precise mechanisms that gp96 enables its substrate maturation and assembly and exerts its function in these biological processes remains largely unclear.

A peptide-based inhibitor of gp96 was previously reported which targets the N-terminal helix-loop-helix sequence of gp96 [14]. The proposed mechanism was that the gp96 inhibitor which mimics the sequence of its helix (aa444-aa480), disrupts intramolecular helix-helix interaction and inhibits gp96 conformational changes that are known to be required for its chaperoning function. This gp96 inhibitor was subsequently found to block the interaction between lipopolysaccharide (LPS) and HSP90, thereby abrogating the cellular response to LPS [15]. We and others have shown that cell membrane gp96 interacts with HER2 and stabilizes HER2 protein in cell membrane, and promotes downstream signaling [9–11]. Based on these studies, we set out to explore the underlying mechanisms of gp96-meidated HER2 dimerization and HER2 signaling by using the gp96 inhibitor for conformational changes. The results could shed light for understanding interaction of gp96 and its substrate HER2 and facilitates the development of gp96-based inhibitors for breast cancer therapy.

## Materials and Methods

### Peptide synthesis

The gp96 inhibitory peptide p37 (LNVSRETLQQHKLLKVIRKKLVRKTLDMIKKIADDKY), the control peptide p40 (LNASQIRELREKSEKFAFQAEVNRMMKLIINSLYKNKEIF), FITC conjugated p37 and biotin conjugated p37 used in this study were chemically synthesized by Jier Biological Company (Shanghai, China). The purity (> 95%) and molecular weight of the peptides were determined by high-performance liquid chromatography and mass spectrometry.

### Cell culture

All cell lines were obtained from Cell Resource Center, IBMS, CAMS/PUMC, China. All cell lines were maintained in RPMI 1640 medium supplemented with 10% fetal bovine serum (Gibco, NY, USA). Cells were grown in a humidified incubator at 37°C under 5% CO_2_ in air.

### Antibodies and other reagents

Trastuzumab was purchased from pharmacy. gp96 antibodies and LAMP-1 antibody were obtained from Santa Cruz Biotechnology (Santa Cruz, CA). The Hsp90α antibody,Hsp90β antibody andα-tubulin antibody were purchased from Bioworld Technology. All other antibodies were purchased from Cell Signaling Technology (Beverly, Massachusetts). The gp96-specific siRNA and control siRNA were designed and synthesized by RiboBio Co., Ltd. (Guangzhou, China). gp96 siRNA was a 25-nucleotide duplexes and had the following sequence: 5’-AAGUUGAUGAACUGAGAGUACUUCC-3’.

### Trypsin protection assay

10 μM of purified gp96 was incubated with 100 μM p37 or the control peptide at 37°C for 15 min. The reaction was acidified with increasing amounts of 0.1 M citrate to achieve a series of pH values from pH 7.4 to 5.0 to induce conformational changes in gp96, and incubated for additional 5 min at 37°C. The solution was then neutralized with 0.5 M Tris (final pH 7.3). 2 μg of trypsin (TPCK [tolylsulfonyl phenylalanyl chloromethyl ketone] treated; New England BioLabs) were added. Digestion was carried out for 1 h at 20°C, and terminated by the addition of 5× SDS sample loading buffer and heated at 100°C for 10min. The extent of gp96 digestion was analyzed by SDS-PAGE.

### Peptide binding assay

Binding of gp96 was quantified by ELISA assay. Briefly, 96-well streptavidin plates (Thermo Fisher Scientific, IL) were coated with the biotinylated peptides. After blocking with 5% skim milk at 37°C, serial concentrations of purified gp96 ranging from 0 to 20 μg/ml were added to each well in 100 μl of binding buffer (20 mM HEPES (pH 7.2), 20 mM NaCl, 2 mM MgCl_2_ and 100 mM KCl) to allow binding for 1.5 h. Afterwards, the plates were incubated with anti-gp96 antibody and the HRP-conjugated secondary antibody. The substrate TMB (3, 3', 5, 5'-Tetramethylbenzidine) was used for detection. The reaction was measured at 450 nm.

### Co-immunoprecipitation

Total cell lysate was incubated with relevant antibodies and precipitated by Protein G Sepharose beads (GE Healthcare, Piscataway, NJ). The immunocomplex was separated on SDS-PAGE for Western blot analysis.

### Indirect immunofluorescence and confocal laser scanning microscopy (CLSM) analyses

Indirect immunofluorescence and CLSM observations were performed as already reported [11]. Images were acquired using a Leica TCS SP2 confocal laser-scanning microscope (Leica Microsystems, Heidelberg, Germany).

### Flow Cytometry

Flow cytometry analysis was performed on cells without fixation and permeabilization as already reported [11].

### Real-time PCR

Total RNA was extracted with Trizol Reagent. Real-time PCR analysis for HER2 and gp96 mRNA levels was performed using the SYBR Green Premix Reagent (Takara Bio Inc., Shiga, Japan). A GAPDH endogenous control was used for normalization.

### Apoptosis assay

Apoptosis assay was performed using Vybrant Apoptosis Assay kit (Invitrogen) according to manufacturer’s instructions. Both apoptotic cells (PI- Annexin V+) and necrotic and late apoptotic cells (PI+ Annexin V+) were counted.

### CCK-8 assay

Cells were trypsinized and seeded in triplicate into 96 well plates. After treatment, 10 μl of the Cell-Counting Kit (CCK)-8 (Dojindo, Kumamoto, Japan) was added into the triplicate wells and incubated for 1 h. Then the absorbance at 490 nm was measured to calculate the numbers of vital cells in each well.

### Animal studies

The study of mice was approved by the Institute of Microbiology, Chinese Academy of Sciences of Research Ethics Committee (permit number PZIMCAS2011001). All animal experiments were performed in strict accordance with institutional guidelines on the handling of laboratory animals. *In vivo* imaging was performed under sodium pentobarbital anesthesia. Mice were sacrificed by cervical vertebra dislocation. All efforts were made to minimize suffering.

Xenograft tumors were established by injection of 1×10^7^ T47D or Bcap37 cells in 200 μl of PBS orthotopically into the flanks of six-week-old female nude mice. The mice were randomly divided into two groups (5mice/group) 15 d post innoculation when tumor mass reached 150 mm^3^ for Bcap37 cells and 300mm^3^ for T47D. p37 were given intravenously (i.v.) twice a week. Tumor growth was measured every 2 days.

### *In vivo* imaging study

*In vivo* imaging experiments were conducted in accordance with a previous report [11]. Briefly, mice were injected subcutaneously with 5×10^6^ SK-BR-3 luc+ cells per mouse. When the tumor volume reached around 5.8×10^6^ phontons mice were treated with p37 peptide. p37 were given intravenously (i.v.) twice a week. Mice under anaesthesia were injected intraperitoneally (i.p.) with 4 mg of luciferin (Promega) in PBS, and images were recorded by the IVIS Imaging System (Xenogen) 15 min after the injection. The bioluminescence images were quantified by Living Image software (Xenogen).

### Statistical analysis

All data are presented as means ± SD, and significance was determined by two-tailed Student’s *t* test. Value of *P* < 0.05 is considered as a significant difference.

## Results

### Conformational changes of gp96 on the plasma membrane are required for gp96-mediated HER2 dimerization

**Fig 1:**
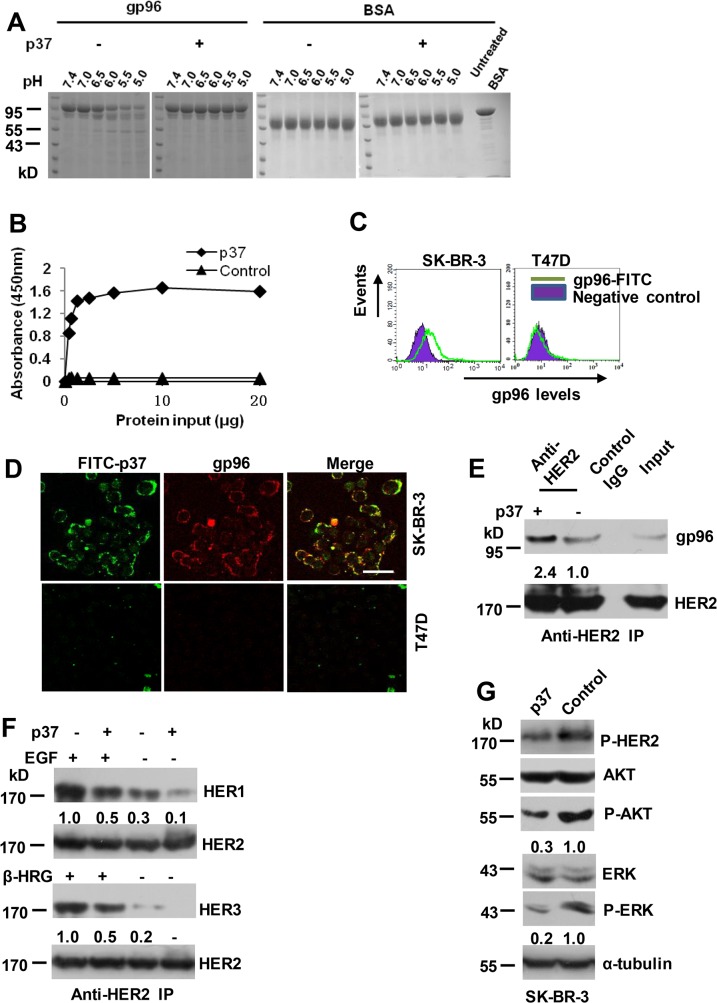
gp96 conformational changes are required for facilitating HER2 dimerization. (A) 10 μM of purified gp96 was treated with 100 μM of p37 (444–480 aa) or control (61–100 aa) peptides and subjected to trypsin digestion at the indicated pH values. BSA served as a control. (B) ELISA analysis of interaction between gp96 and p37 or control peptides. (C) Flow cytometric analysis of cell surface levels of gp96. (D) SK-BR-3 and T47D cells were cultured in presence of FITC-labeled p37 for 30 minutes, and then stained by immunofluorescence (TRITC) using an anti-gp96 antibody. Scale bar, 40 μm. (E-F) SK-BR-3 cells were treated with 20 μg/ml of p37 or control peptide for 30 min. Cell lysates were immunoprecipitated with anti-HER2 antibody (E). SK-BR-3 cells were pre-treated with EGF (50 ng/ml) or β-heregulin (100 ng/ml) for 15 min, and then cells were treated and analyzed as in E (F). Numbers below the blot indicates quantification shown on Western blot after normalization to HER2. (G) Western blot assay of cell lysates of SK-BR-3 cells treated with 20 μg/ml of p37 or control peptide for 8 h. The ratios of P-AKT to AKT and P-ERK to ERK were calculated, and the values were shown. The ratios in control peptide-treated cells were arbitrarily taken as 1.0.

The gp96 inhibitory peptide derived from an α-helix in the middle domain of gp96 (444–480 aa, designated as p37) has been shown to block intramolecular conformational changes that involves the formation of helix-helix interaction [14]. A trypsin-protection assay was used to further confirm p37 peptide inhibition of conformational change of gp96. The results showed that in contrast to the control peptide (corresponding to 61–100 aa of gp96)-treated gp96 that was progressively digested between pH 6.5 to pH 5.0, p37 peptide-treated gp96 was resistant to trypsin digestion (Fig 1A). The p37 peptide bound to gp96 in a dose-dependent fashion until a saturation level was reached, while the control peptide did not bind gp96 (Fig 1B). High level of cell membrane gp96 expression was observed in SK-BR-3 cells, whereas low or no cell membrane gp96 was detected in T47D cells by FACS analysis (Fig 1C). Confocal microscopy analysis showed a nearly perfect colocalization of gp96 and FITC-conjugated p37 peptide prominently at the cell membrane on SK-BR-3 cells (Fig 1D). In contrast, only faint immunofluorescence staining was observed in T47D cells, validating specific target recognition of the gp96 inhibitor.

We next determine if the conformational changes of gp96 affect the association between gp96 and HER2. Treatment with p37 peptide quickly led to a significant increase in the amount of gp96 associated with HER2 by the coimmunoprecipitation assay (Fig 1E), which subsequently resulted in abrupt suppression of HER2 dimerization with HER1 and HER3 (Fig 1F), and the HER2 phosphorylation and downstream signaling pathways (Fig 1G). Together, these data suggest that the gp96 conformational change inhibitor p37 exerts blocking effect on HER2 heterodimerization with other HER family members probably by preventing release of HER2 heterodimers from gp96 molecules.

### Blockage of gp96 conformational changes induces HER2 ubiquitinylation and lysosomal degradation

**Fig 2:**
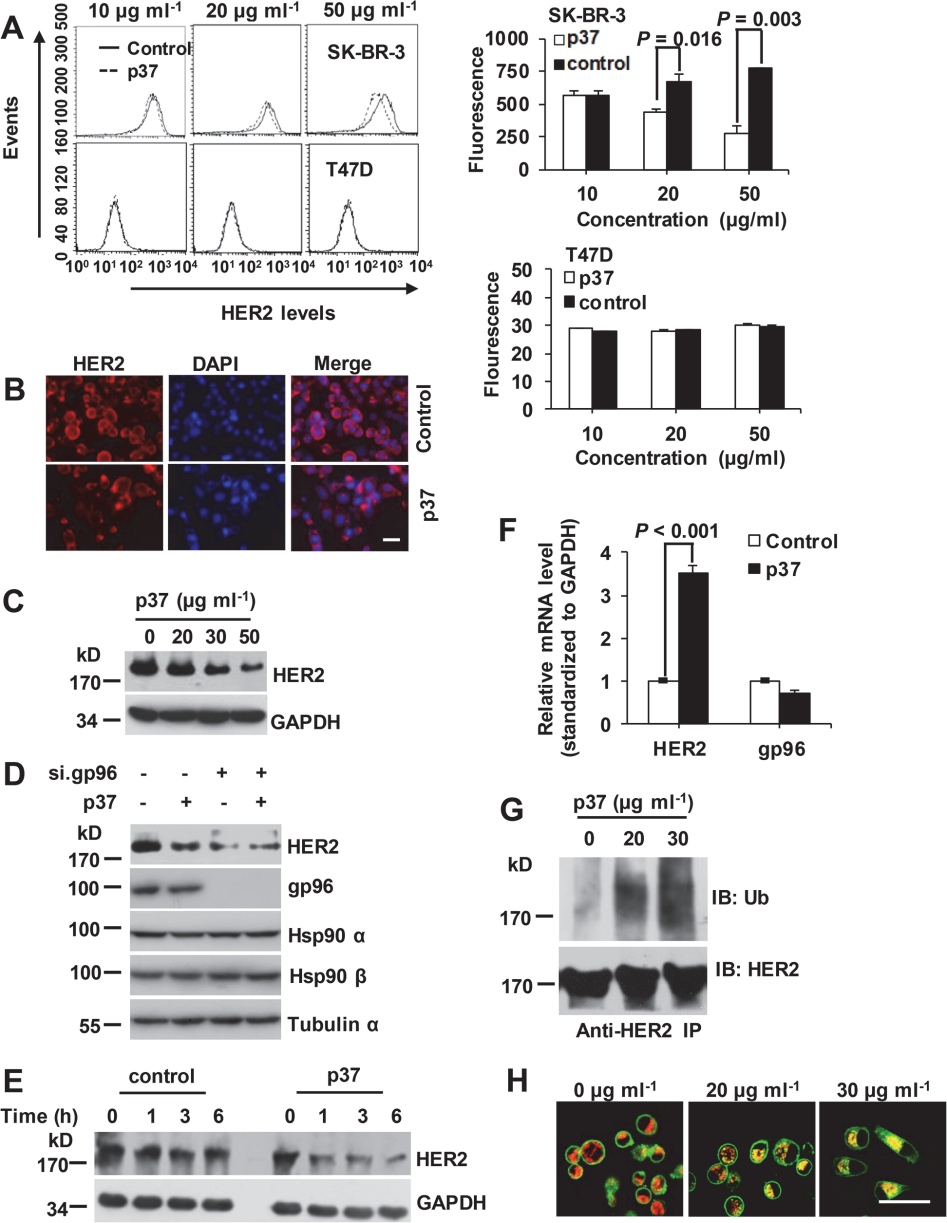
p37 peptide treatment induces HER2 degradation. (A) Flow cytometric analysis of HER2 levels in SK-BR-3 cells treated with the indicated concentration of p37 for 8 h. HER2-low expressing T47D cells served as a control. (B) IF staining of HER2 in fixed and permeabilized SK-BR-3 cells treated with 20 μg/ml of p37 for 8 h. Scale bar, 40 μm. (C) Immunoblot detection of HER2 in SK-BR-3 cells treated with the indicated concentrations of p37 for 8 h. (D) SK-BR-3 cells were transfected with gp96 or control siRNA for 48 h. Cells were then treated with 20 μg/ml of p37 or control peptide for additional 8 h. Cell lysates were subjected to Western blotting. Hsp 90α, Hsp90β and α-tubulin served as controls. (E) Immunoblot detection of HER2 in SK-BR-3 cells treated with 20 μg/ml of p37 or control peptide along with 100 μg/ml of cycloheximide for the indicated times. (F) Real-time PCR analysis of gp96 and HER2 mRNA levels in SK-BR-3 cells treated with p37 or control peptide. (G) SK-BR-3 cells were incubated with 50 μM MG-132 and the indicated concentrations of p37. HER2 was immunoprecipitated and subjected to immunoblot with anti-ubiquitin antibody. (H) SK-BR-3 cells grown on coverslips were treated with 100 nM Bafilomycin A1 for 1 h. Cells were then treated with the indicated concentrations of p37. Cells were then fixed and stained for HER2 (green) and LAMP-1 (red). Scale bar, 40 μm. Results are presented as means ± SD from three independent experiments.

Next, we assessed the effect of p37 peptide on HER2 level. In accord with gp96 inhibition by small molecules and gp96 monoclonal antibodies [10,11], treatment with p37 peptide in SK-BR-3 cells led to depletion of cell surface expressed HER2 by FACS analysis (Fig 2A) and total HER2 determined by immunofluorescence staining (Fig 2B) and Western blotting assay (Fig 2C), respectively. No significant changes of HER2 were observed in p37-treated T47D cells with low level of HER2 expression (Fig 2A, lower panel). Moreover, the effect of p37 on HER2 was largely abolished by knocking down of gp96 by RNAi (Fig 2D), indicating that p37 affects HER2 level mainly via gp96. A sharp time-dependent decrease of HER2 protein was observed in p37-treated cells but not in control cells in the presence of cycloheximide (CHX) (Fig 2E), whereas HER2 mRNA levels were increased in p37-treated cells probably due to compensation of the loss of HER2 (Fig 2F), indicating that p37 caused a decrease of HER2 protein stability. p37 peptide treated cells showed an increase in HER2 ubiquitination (Fig 2G). Under p37 peptide treatment, HER2 was colocalized with the lysosome marker LAMP-1, while untreated cells showed almost no HER2 and LAMP-1 colocalization (Fig 2H).

### Sensitivity of breast cancer cells to gp96 inhibitory p37 peptide is associated with cell membrane gp96 and HER2 levels

**Fig 3:**
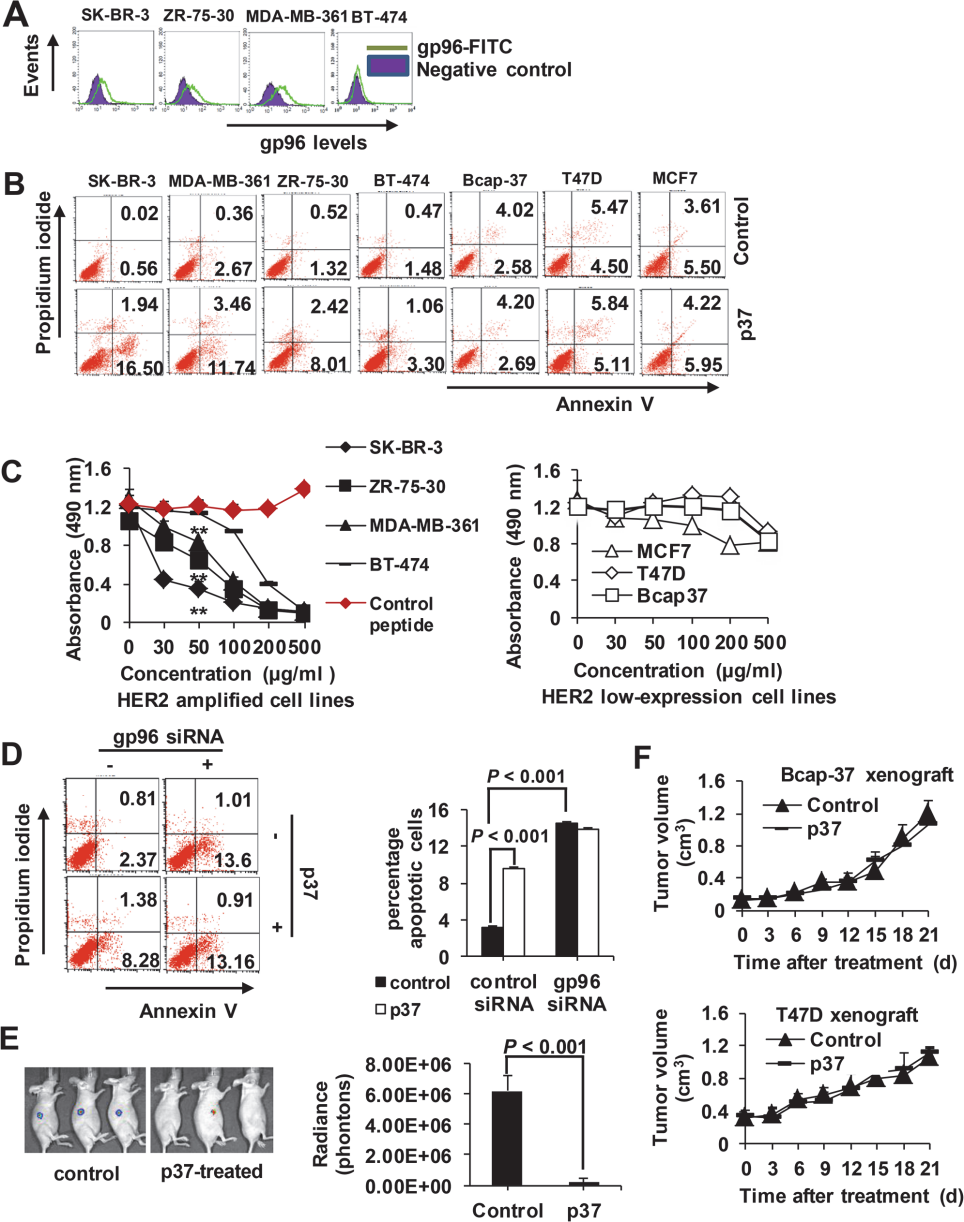
p37 peptide suppresses cell growth and induces apoptosis in breast cell lines. (A) Flow cytometric analysis of cell surface gp96 levels in breast cancer cells. (B) Cells were treated with 50 μg/ml of p37 for 48 h. Cellular apoptosis were analyzed by FACS, and the percentage of apoptotic cells (Annexin V single positive and Annexin V/PI double positive) was determined. (C) CCK-8 assay of cell proliferation upon treatment with various concentrations of p37 peptide for 48 h. Results are presented as means ± SD from three independent experiments. (D) SK-BR-3 cells were transfected with gp96 or control siRNA for 48 h. Cells were treated with 20 μg/ml of p37 or control peptide for additional 48 h. Cellular apoptosis were analyzed by FACS. Results are presented as means ± SD from three independent experiments. (E) Representative *in vivo* luciferase images of SK-BR-3 xenograft nude mice at day 21 after p37 (8 mg/kg) treatment. (F) T47D and Bcap-37 xenograft tumor volume in response to p37 (8 mg/kg) treatment. Data shown are the means and SDs of five mice. Data are representative of two independent experiments. **, *P* < 0.01 compared with controls.

In this study, SK-BR-3, BT-474, ZR-75-30 and MDA-MB-361 cells were used as HER2 amplified cell lines, and Bcap-37, T47D and MCF7 cells were used as HER2 low expressing control cell lines [16]. High levels of cell membrane gp96 were detected in SK-BR-3, ZR-75-30 and MDA-MB-361 cells among HER2 amplified cells (Fig 3A). Annexin V/PI staining showed that under p37 peptide treatment, the cell apoptosis rate strongly correlated with the membrane gp96 and HER2 levels in these breast cancer cells (Fig 3B). p37 peptide at 50 μg/ml induced more pronounced apoptosis in gp96 highly expressed and HER2 amplified cells than in gp96 or HER2 low expressing cells (S1 File). Similarly, CCK-8 assay showed that p37 peptide caused the most evident decrease of cell proliferation in SK-BR-3, ZR-75-30 and MDA-MB-361 cells with high gp96 and HER2 expression (*P* < 0.01 vs. BT474 and HER2 low expressing cells) (Fig 3C), while no effect was observed for the control peptide. As seen in Fig 3D, depletion of gp96 largely abolished p37-induced cell apoptosis. *In vivo* imaging showed that p37 peptide treatment (8 mg/kg) dramatically decreased SK-BR-3 tumor size 20 d after treatment (*P* < 0.001, Fig 3E). In contrast, p37 peptide had limited effects in HER2 low expressing tumor models (Fig 3F). These results indicate that the effects of gp96 inhibition on breast cancer are dependent on cell membrane gp96 and HER2 status.

### p37 peptide synergizes with trastuzumab to suppress cell growth and induce apoptosis

**Fig 4:**
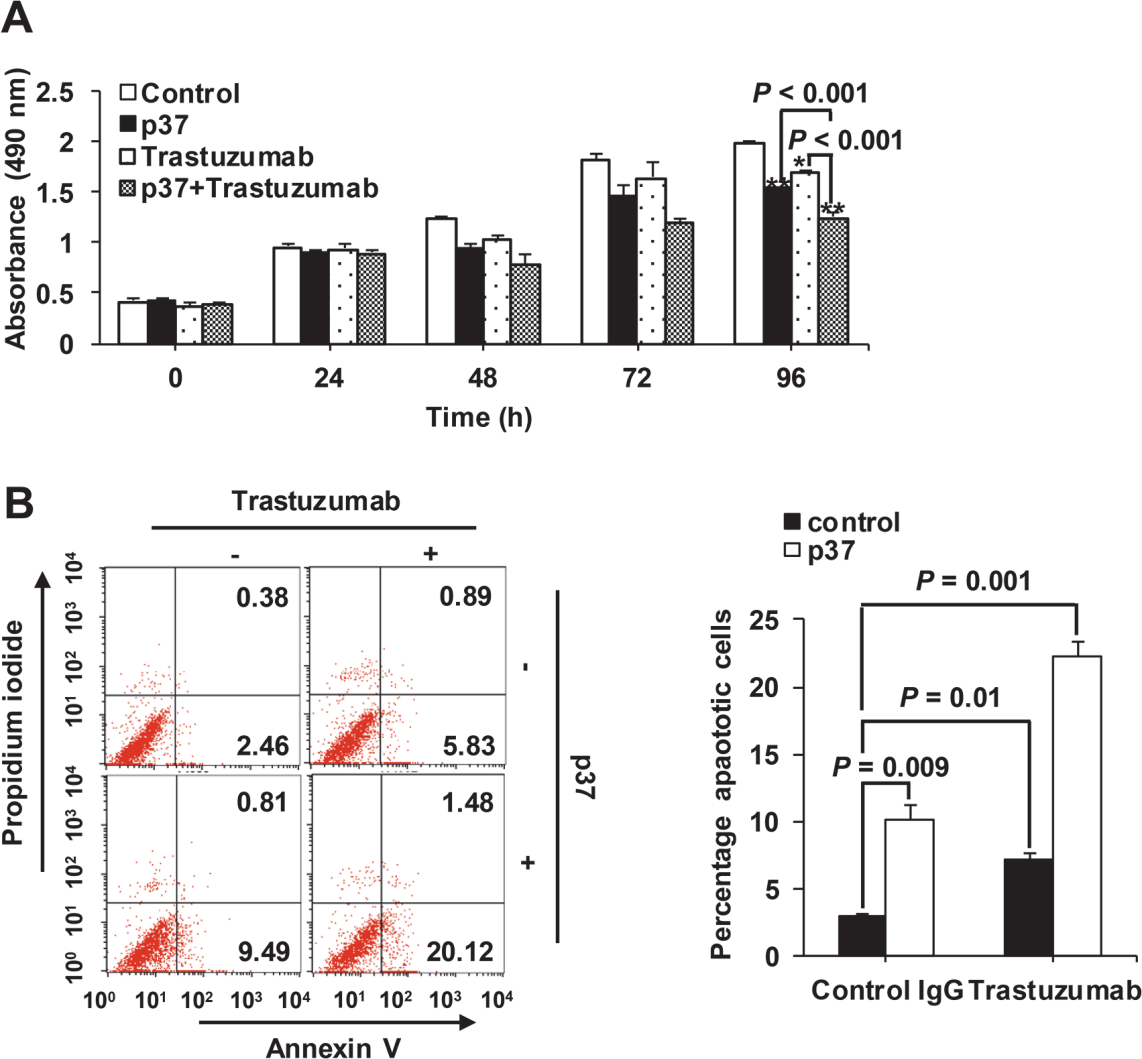
p37 peptide synergizes with trastuzumab to suppress cell growth and induce apoptosis. (A) SK-BR-3 cells were treated with 20 μg/ml of p37 or control peptide, and 20 μg/ml of trastuzumab for the time as indicated. Cell proliferation was measured by CCK-8 assay. (B) Cell apoptosis was analyzed by flow cytometry, and the percentage of apoptotic cells (Annexin V single positive and Annexin V/PI double positive) was determined after p37 peptide treatment for 72 h. Error bars indicates the SDs of three independent experiments. *, *P* < 0.05 compared with controls; **, *P* < 0.01 compared with controls.

We further investigated if p37 peptide which inhibits HER2 heterodimerization, has a mechanism of tumor cell growth-inhibitory action that is complementary to that of trastuzumab which has been shown to preferentially block HER2 homodimer [17]. The results showed that either p37 or trastuzumab treatment suppressed cell growth (Fig 4A) and induced cell apoptosis (Fig 4B), and notably these effects were significantly enhanced when treated with both p37 and trastuzumab (all *P* < 0.01).

## Discussion

HER2 receptor dimerization is an essential step for the HER2 activation process. However, little attention has been paid to the regulation of HER2 dimerization for understanding the mechanism of encountering and subsequent pairing of HER receptors on the plasma membrane. In the present study, we found that inhibition of gp96 intramolecular conformational change causes increased binding of gp96 to HER2 and decreased HER2 heterodimerization. Moreover, we have demonstrated that blocking gp96 conformational changes directs HER2 internalization and lysosomal and proteasomal proteolysis, and inhibits HER2-driven cell growth.

Our current data, combined with previous studies showing that cell membrane gp96 N-terminal domain was able to interact with the HER2, and facilitates HER2 dimerization and promotes its downstream signaling [9–11], along with the seminal finding by Dollins *et al* that the conformational changes of gp96 occur from a twisted extended-open dimer to transient closed dimer form between the two N-terminals in ATP hydrolysis [18], demonstrate the possible mechanism of gp96-mediated HER2 dimerization by binding and bringing HER2 and its partner to contact distance, or/and inducing conformational changes of HER2 to promote its ability to form dimers. According to our model, gp96 dimer in “extended-open” conformation upon ATP binding spontaneously associates with HER2 or other HER members through gp96 N domain. The conformational switch to the hyrolytically productive “closed” state by the dimerization between the two N domains of gp96 brings HER2 and its partners into close vicinity, or/and induces HER2 in a conformation that allows dimerization, thereby facilitating HER2 dimer formation and functional activation. There have been evidences supporting our hypothesis. gp96 exists as a homodimer, and adopt twisted V ground-state conformation for client protein interactions [18]. The gp96 inhibitor p37 binds to the middle domain of gp96 and blocks rotations of the N-terminal-middle domain junction [14,18], thus may prevent conformational switch of gp96 dimer from the twisted V “open” state to the N-terminal dimerized “close” state. In accordance with the working hypothesis, p37 treatment induced dramatic increase of HER2 bound to gp96 and decreases of HER2 dimers, suggesting that HER2 dimerization and release from gp96 occurs after gp96 undergoes conformational changes to “closed” state. These studies support the pivotal role of gp96 in HER2 signaling.

There is now emerging evidence that gp96, as a pro-oncogenic chaperon for multiple onco-proteins, such as HER2 [9–11], the Wnt coreceptor LRP6 [19,20], integrin [5], toll like receptors [21], p53/Mdm2 [22], plays important roles in regulation of cancer initiation, progression and metastasis. In addition, gp96 knockout in liver predisposes mice to develop carcinogen-induced liver cancer, indicating that gp96 is an attractive therapeutic target for cancer [8]. In contrast to gp96-specific small inhibitory molecules and gp96 monoclonal antibodies which disrupt the binding of gp96 with HER2 [10,11], the gp96 conformational change inhibitor p37 led to increased rather than decreased association between gp96 and HER2. We hypothesize that gp96-mediated HER2 dimerization can be divided into two distinct steps. First, gp96 binds HER2 and possibly other HER membranes. Second, gp96 molecules undergo conformational changes and induces HER2 dimerization, and the formed HER2 dimers releases from gp96 molecules. The gp96-specific small inhibitory molecules and gp96 monoclonal antibodies may target the first step whereas the gp96 conformational change inhibitor p37 possibly targets the second step. This deserves further investigation.

HER2 signaling is preceded by receptor dimerization, so it is worthwhile to explore the regulation of interconversion between monomer and dimer state of HER2 and other HER members, and the formation of HER2 homodimers or heterodimers under different physiological and pathological conditions. The results presented here suggest that cell membrane gp96 mediates HER partners’ engagement by conformational changes, resulting in the formation of HER2 dimers. Our study presents an effort to address the mechanism of collisional encounter of receptors and subsequent pairing of HER receptors mediated by gp96. Our results indicate that cell surface gp96 may work as a scaffolding protein linking HER2 and its dimerziation partners to pair. Inhibition of conformational changes of plasma membrane gp96 may affect the release of HER2 heterodimers from gp96. The results may also provide bases for designing and evaluating effective peptide-based inhibitor against cell membrane gp96, and help to develop a novel therapeutic approach for inhibiting HER2 signaling in cancer.

## Supporting Information

S1 File Supporting Data. (DOC)
