# Supplementary material for: Blockage of Conformational Changes of Heat Shock Protein gp96 on Cell Membrane by a α-Helix Peptide Inhibits HER2 Dimerization and Signaling in Breast Cancer
Source: PLoS One. 2015 Apr 21;10(4):e0124647. doi: 10.1371/journal.pone.0124647 (PMC4405268; doi:10.1371/journal.pone.0124647)
Supplement: S1 File — (DOC) [file pone.0124647.s001.doc]

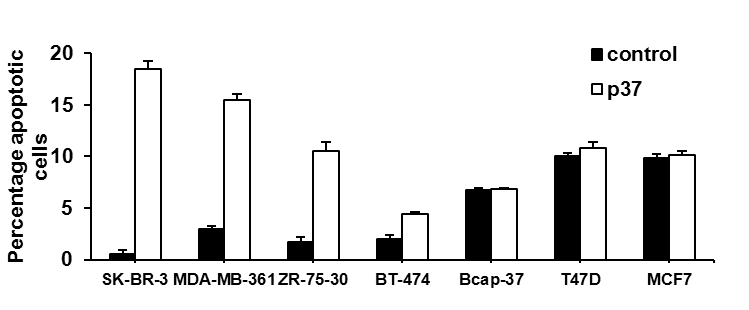


***P <* 0.001**

***P <* 0.001**

***P <* 0.001**

***P =* 0.015**

**Supplementary Figure S1. | p37 induced cell apoptosis correlated with membrane gp96 and HER2 levels in breast cancer cells.** Cells were treated with p37 peptide as in Fig. 3B. Cellular apoptosis were analyzed by FACS, and the percentage of apoptotic cells (Annexin V single positive and Annexin V/PI double positive) was determined. Results are presented as means ± SD from three independent experiments.
